# National Medical Convention

**Published:** 1847-02

**Authors:** 


ARTICLE XVII.

### NATIONAL MEDICAL CONVENTION.

We are pleased to see this proposition for the improvement of the profession meeting with more general approbation. Most of our cotemporaries have spoken in its favor, and many of the most respectable societies and colleges of the United States have appointed delegates to the meeting, to be held at Philadelphia on the first Wednesday in May next.

That some of the plans for reform in medical education proposed for the adoption of this Convention are utopian in the extreme, must be apparent; yet we see no reason why great good may not result from the deliberations of such a body of physicians, made up as it should be of the representatives of the great body of the profession in the United States; and as the influence of the Convention must to a great extent depend upon the fullness of the representation, we hope that all bodies entitled by the rules adopted will be represented on the occasion. E.

